# Liver involvement in juvenile systemic lupus erythematosus (SLE)

**DOI:** 10.1186/1546-0096-9-S1-P254

**Published:** 2011-09-14

**Authors:** Generosa Mariniello, Giustina Russo, Raffaella Carlomagno, Rosalba Vitale, Maria Alessio

**Affiliations:** 1 Rheumatology Unit, Department of Pediatrics, Federico II University, Naples, Italy

## Introduction

Abnormal liver tests are common among adult patients with systemic lupus erythematosus (SLE). The most common cause is drug-induced hepatitis, while mild, predominantly lobular, but sometimes also portal and periportal-hepatitis reflecting SLE activity, is another possibility. Little is known about liver disease in juvenile SLE patients although mild transient liver enzyme abnormalities are noted in up to 25%.

## Aim

to assess the frequency of liver involvement in a cohort of pediatric patients with SLE.

## Methods

We carried out a retrospective chart review of 32 patients with SLE, including demographic data, clinical features, laboratory findings, and treatments, as well as outcomes. Liver enzyme values were considered abnormal when a sustained increase in serum transaminase levels above the normal value was observed for a period of at least three months or when the increase was confirmed in two consecutive assessments. SLE patients with elevated liver enzymes were investigated for liver diseases.

## Results

Liver involvement was noted in 6 patients (18.75%): aminotransferases exceeded 1.5 times the normal values. The ultrasound showed a liver increased in size in 2 patients. In one patient liver involvement was concurrent with the diagnosis of SLE and associated with thrombocytopenia, aminotransferases normalized with the beginning of prednisone. No patient fulfilled criteria for the diagnosis of autoimmune hepatitis.

## Conclusions

In 5/6 patients (83%) liver dysfunction was probably drug induced. The two children with hepatomegaly didn’t fulfilled scoring system’s criteria for autoimmune hepatitis. In this study we have a lower incidence of liver dysfunction.

